# Association among multimorbidity, physical disability and depression trajectories: a study of urban–rural differences in China

**DOI:** 10.1007/s11136-021-02807-3

**Published:** 2021-03-06

**Authors:** Chaoyang Yan, Hui Liao, Ying Ma, Qin Xiang, Jing Wang

**Affiliations:** 1 Department of Health Management, School of Medicine and Health Management, Tongji Medical College, Huazhong University of Science and Technology, Wuhan, 430030 Hubei China; 2 The Key Research Institute of Humanities and Social Science of Hubei Province, Huazhong University of Science and Technology, Wuhan, 430030 Hubei China; 3 Institute for Poverty Reduction and Development, Huazhong University of Science and Technology, Wuhan, 430030 Hubei China

**Keywords:** Multimorbidity, Physical disability, Trajectory, Depression

## Abstract

**Purpose:**

The purpose of this study was to analyse the trajectories of depression in urban and rural areas, and to analyse the relationship among multimorbidity, disability and other variables and trajectories.

**Methods:**

Data from the China Health and Retirement Longitudinal Study were used. A latent class growth model was used to characterise the trajectories of urban and rural depression symptoms. Chi-square test was used to test the differences in respondents’ characteristics among depression trajectories groups within urban and rural areas. The relationships among multimorbidity, disability and depression symptom trajectories were analysed via multinomial logistic regression.

**Results:**

Urban and rural depression trajectories were divided into three categories. Respondents in urban areas were divided into rising, remaining-low and declining group, and those in rural areas were divided into rising, remaining-low and remaining-high group. The depression scores of respondents with multimorbidity were more likely to rise, and this result was similar for the disabled respondents. Respondents who need help on activities of daily living and instrumental activities of daily living in urban areas were more likely to decline in depression scores. In rural areas, however, the values were consistently high. In urban and rural areas, the relationships among marital status, education and age and depression trajectories were different.

**Conclusions:**

The depression trajectories are different in urban and rural China. Improving the quality of medical services, promoting the distribution of rural social resources and implementing more recreational activities could be beneficial for the promotion of mental health in rural areas.

## Introduction

Depression is very common and has become a growing public health concern, affecting more than 300 million people of all ages and causing up to 43.1 million all-age disability-adjusted life years (DALYs) [1, 2]. The overall estimation of current, 12-month and lifetime prevalence of major depressive disorder in China is 1.6%, 2.3% and 3.3%, respectively [3]. A meta-study indicated that the pooled prevalence of depression symptoms was 23.6% (95% confidence interval 20.3–27.2%) among the elderly in China [4].

Research on depression is primarily concerned with influencing factors, prevention, treatment and financial burden. A ‘U’-shaped curve was demonstrated between body mass index and post-stroke depression in patients aged less than 65 years [5]. The relationship between juvenile delinquency and adult depression was also investigated. Opie et al. focused on the relationship between dietary patterns and specific dietary factors and the prevention of depression [6]. Pastoor et al. have discussed medications for depression [7]. Cuijpers et al. pointed out that psychotherapy is effective in the primary care of depression and has a more lasting effect than drugs [8]. Greenberg et al. illustrated that the economic burden of patients with major depressive disorder in the United States increased by 21.5%, and the composition of these costs remained stable, with about 45% attributed to direct expenses [9]. Due to the differences in China's urban and rural economic development level, household registration system design and employment types (rural population is mainly engaged in agricultural activities, while urban population is mainly engaged in industry and service), there are great differences in income, social resource allocation and access to welfare policies between urban and rural areas [10–12]. In 2018, the per capita disposable income of urban residents in China was 39,251 yuan, and that of rural residents was 14,617 yuan, which is a large gap. In 2015, 47.1% of the rural population in China had an education level of primary school or below, which was much higher than the 13.1% in urban [13, 14].

Many studies have also been performed regarding the trends in depression. The incidence of depression increased by 49.86% from 1990 to 2017 worldwide [15]. From 1990 to 2017, in China, the all-age prevalence rate of depression per 100,000 rose from 3224.6 (95% uncertainty interval (UI): 2976.6–3509.1) to 3990.5 (95% UI: 3667.8–4353.0) [16]. The DALYs caused by depression in China is estimated to increase by about 10% from 2013 to 2025 [17]. Hence, the depression symptoms of the population may change over a period of time.

Several studies have paid attention to the trajectory of depression and its influencing factors. Saunders et al. analysed the trajectory category of depression and anxiety symptoms during psychotherapy and the baseline patient characteristics related to the trajectory [18]. Leung et al. analysed the relationship between chronic disease at baseline and the trajectory of depression [19]. Studies should also compare the trajectories of depression symptoms over time among different populations and analyse the relevant factors.

The trajectory of depression in urban and rural areas in China may be different because the prevalence of depression and the allocation of social resources vary between them. Zhang et al. pointed out that the prevalence of depression among the elderly in rural areas (29.2%) is significantly higher than that of the urban population in China (20.5%) [20]. Huo et al. described evident differences between urban and rural welfare systems in China [21]. Liu et al. explained that urban areas have more government support than rural areas in China [22]. Chen et al. demonstrated the differences in the distribution of health resources between urban and rural areas in China [23].

Physical health status, including multimorbidity and physical disability, can be tightly related to the trend of depression. According to a survey conducted among 5296 participants in East China, those with multiple chronic diseases were likely to have a depression symptom [24]. In addition, a British study showed that concurrent sensory disorders are associated with a low quality of life and high risk of depression symptoms [25]. Beck’s model of depression development suggests that depression symptoms include a negative self-interpretation of an individual’s multiple comorbidities [26]. Quality of life is a multi-dimensional concept. The study focused on the relationship between physical and mental health dimensions and took into account time changes, which has important implications for dynamic interventions to improve quality of life.

Additionally, socioeconomic status (SES) may affect the changes in depression. Women are more likely to have depression than men [27, 28]. Age is also associated with depression symptoms [29]. Meanwhile, married people have better mental health than those who are single, widowed, separated and divorced [30]. Low levels of education and income are risk factors for depression [31]. The relationship between working hours and depression has also been previously demonstrated [32]. Compared with living with a spouse alone, individuals living with a spouse and a child or those living alone were more likely to have depression symptoms [33]. Among psychiatric patients, smoking is associated with severe symptoms of depression and anxiety and with slow recovery but only in the case of nicotine-dependent smokers. A J-shaped relationship has been confirmed between alcohol consumption and depression among elderly people in South Korea [34].

Considering the factors related to depression symptoms can probably influence the depression development trend. The kinds of factors associated with the trajectory of depression development must be studied. Furthermore, remarkable differences exist in resource allocation and welfare system between urban and rural areas in China, and such differences can be closely related to depression prevention and control. Thus, a comparative study should be conducted on the trajectory of depression, especially, its influencing factors in urban and rural areas.

This study aimed to (1) explore the differences in depression trajectory between urban and rural areas in China with three-wave panel data; (2) find associated factors of the depression trajectory by analysing the relationship between physical health status, including multimorbidity and physical disability and other independent variables at baseline, and depression trajectories.

This study will be helpful to support depression prevention, and controlling dynamically. The research on the trajectory and influencing factors of depression in China, as the largest developing country in the world, can be meaningful to reveal the relationship between social resource allocation and mental health, especially for developing or underdeveloped areas of the world.

## Methods

### Data sources

The data used in this study were obtained from China Health and Retirement Longitudinal Study (CHARLS), targeting the middle-aged and older population (45+ years), in China. CHARLS is an ongoing large-scale interdisciplinary investigation project sponsored by the National Development Research Institute of Peking University and jointly implemented by the Chinese Social Sciences investigation centre of Peking University and the Youth League Committee of Peking University, with surveys performed every 2 years from 2011 to 2015.

CHARLS is sampled at the county-village-household-individual level through four stages. Specifically, in county-village sampling, CHARLS adopts probabilities that are proportional to size sampling. Based on the population of each district and county in 2009 and using regional, urban and rural areas and gross domestic product as stratified indicators, 150 counties (districts) were randomly selected in the whole country. Three villages (communities) were randomly selected from each of the 150 counties (districts) based on the permanent population of each village or community in 2009. A total of 450 villages (communities) were finally obtained. Exactly 80 households were randomly selected from the information list of all households in each sample village (communities).

**Fig. 1:**
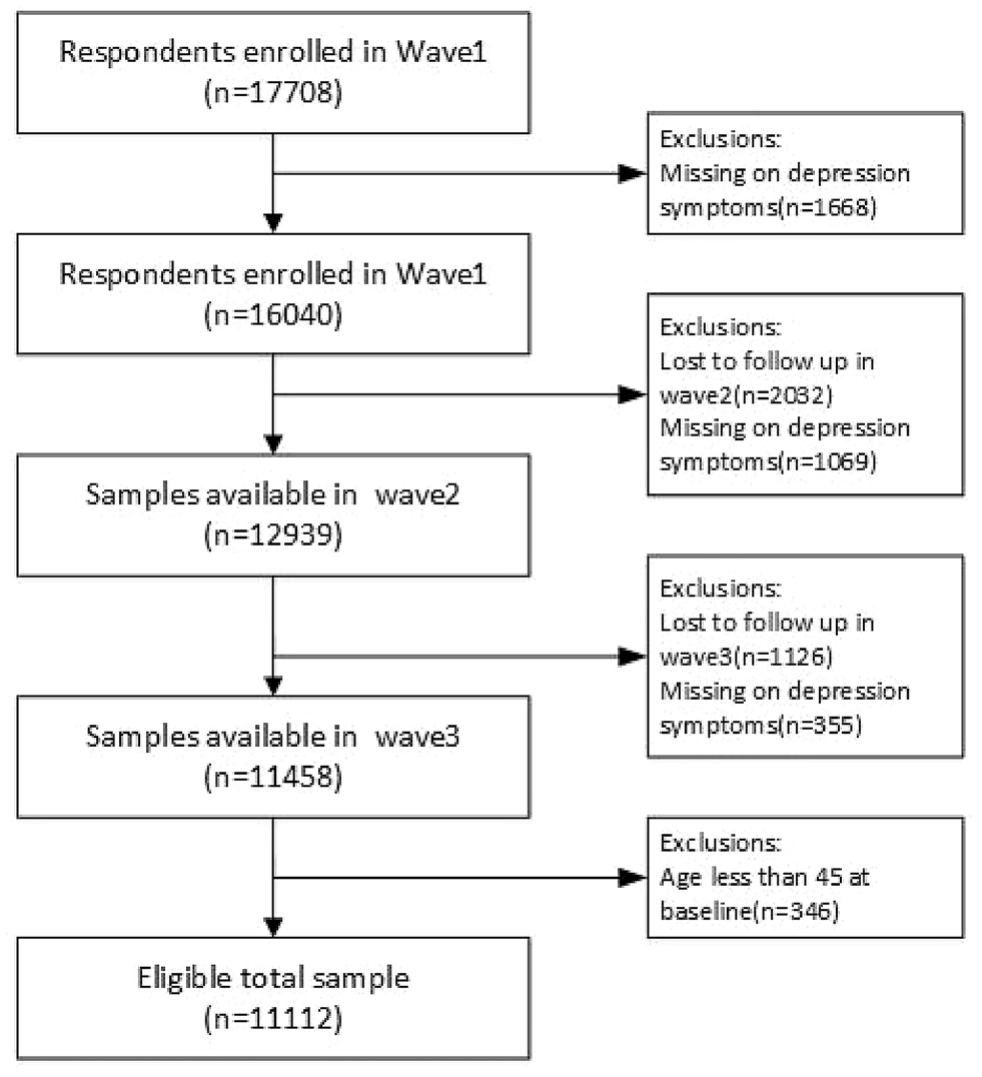
Flowchart of sample selection

The main household questionnaire of CHARLS contains information regarding basic demographics, health status, healthcare, employment and household economy (income, consumption and wealth). The national baseline survey of CHARLS was conducted between June 2011 and March 2012 on 17708 respondents. After excluding the depression missing data and samples that were lost to follow-up, 11,112 samples from three-wave panel data were analysed in this study (Fig. 1). Depression data were calculated in each wave, and multimorbidity, physical disabilities and SES and other independent data were all from wave 2011 as the baseline.

### Variables and definitions

#### Depression

Depression was decided by the respondents’ positive feelings, negative emotions and somatic symptoms in the CHARLS questionnaire. The 10-item Center for Epidemiologic Studies Depression Scale (CES-D10) was used to describe the depression symptoms, and the responses for CES-D10-based questionnaire were coded on a four-point scale from 1 to 4. Hence, the total scores for depression symptoms ranged between 10 and 40, with 10 representing a perfect healthy mental status and 40 reflecting extremely severe depression. In this study, depression was used as a continuous variable based on the CES-D10 scores.

#### Physical health status

Respondents of CHARLS were asked to report chronic diseases since wave 2011. The fourteen kinds of chronic diseases should be diagnosed by doctors and ongoing for at least half a year. In this study, multimorbidity was defined as a binary variable. Thus, one group had one or none of the kinds of chronic diseases, and the other included two or more types of chronic diseases. Quality of life is a multi-dimensional concept. Its scope is larger than the state of physical health. However, physical health status is a part of quality of life. Our research focuses on reflecting quality of life through physical health status.

In this study, physical disability was evaluated based on activities of daily living (ADL) and instrumental activities of daily living (IADL). The CHARLS questionnaire in 2011 contained six ADL items (dressing, bathing, eating, getting into and out of bed and toileting, controlling urination and defecation) and five IADL items (preparing a hot meal, shopping for groceries, making telephone calls, taking medicines and managing money). Given that ADL and IADL represent different aspects of disability [35, 36], the study classified physical disability into four groups: neither ADL nor IADL needs help, only need help on IADL, only need help on ADL, both ADL and IADL need help.

#### Other independent variables

The SES variables analysed in this study included gender, age, marital status, education, occupation status, family income, living arrangement and lifestyle. Age was divided into four subgroups, namely, 45–49, 50–59, 60–69 and 70 years or above. This study defined marital status as a binary variable, implying that married couples who lived with their spouse or temporarily did not live together as having a spouse, whereas other marital conditions were treated as not having a spouse. Education in this study was divided into four levels: illiterate, primary school, junior high school and senior high school and above. Given that the respondents of CHARLS were mostly over 45 years old, we separated them into those who are still employed or engaged in work-related activities from all the respondents as occupation status. Household income per capita was used to measure the economic status of the respondent’s family, and quintile was used to show their economical level. Furthermore, based on whether the respondents lived in the same town or city as their children, the respondents’ living arrangements were divided into two groups. In addition, smoking and drinking were included in the study and classified by lifestyle of smoking or drinking in the year before the survey. Table 1 provides the detailed coding of each variable.

**Table 1:** Coding of physical health status variables and other independent variables

Variable	Codes
Physical health status	
Multimorbidity	1=no, 2=yes
Physical disability	1=no limits, 2=only IADL, 3=only ADL, 4=both
Other independent variables	
Gender	1=male, 2=female
Age (years)	1=45–49, 2=50–59, 3=60–69, 4=≥70
Marital status	1=partnered, 2=single
Education	1=illiteracy, 2=primary school, 3=junior high school, 4=senior high school and above
Occupation status	1=yes, 2=no
Family income	1=worst, 5=best, from 1 to 5 is from the best to the worst
Smoke	1=yes, 2=no
Drink	1=yes, 2=no
Living arrangement	1=yes, 2=no

### Statistical method

The latent class growth model, which is a model used to describe the development feature of samples in a certain period and classify the development feature, was used to illustrate and classify the developmental trajectories of urban and rural depression symptoms in this study. Small values of Akaike Information Criterion (AIC), Bayesian Information Criterion (BIC) and adjusted Bayesian Information Criterion (aBIC) are preferred in selecting the number of latent classes [37]. After determining the trajectory groups, Chi-square analysis was used to test the differences in respondents’ characteristics among depression trajectories groups within urban and rural areas. Then, multinomial logistic regression was used to explore the association between trajectories of depression symptoms and baseline multimorbidity, physical disability and other independent variables in urban and rural areas.

Mplus 8.0 was used to apply the latent class growth model, and STATA software 13 was used to execute the multinomial logistic regression and Chi-square analysis, with statistical significance at *p* < 0.05.

### Ethical approval

Ethical approval for data collection in CHARLS was obtained from the Biomedical Ethics Review Committee of Peking University (IRB00001052–11015). All interviewees gave written informed consent before recruitment to the study.

## Results

**Fig. 2:**
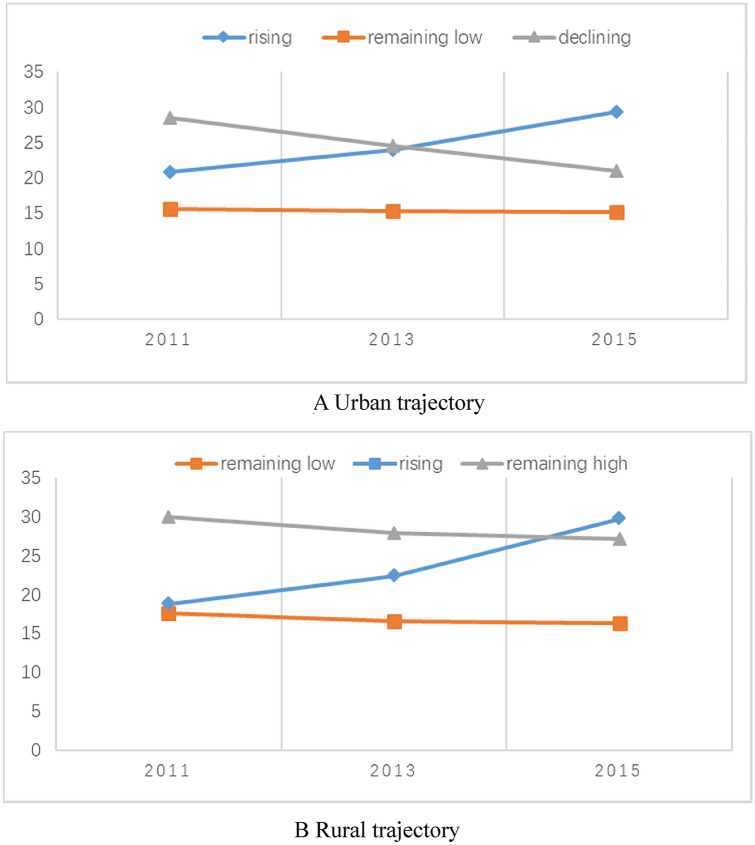
The trajectory of depression symptom in urban and rural. **a** Urban trajectory, **b** rural trajectory

Table 2 shows the model fitting results of the latent class growth model for depression symptoms. Panels A and B represent the model fitting results of urban and rural areas, respectively. We classified the trajectories of depression in urban and rural areas into three groups each, in accordance with the criteria including small values of BIC, aBIC and AIC and an entropy score close to 1 [38]. According to the estimated posterior probabilities, the proportions of urban areas divided into three groups were 79%, 11% and 10%, and those of rural areas were 77%, 12% and 11%. Very small classes (< 10% of the total sample) may represent an accidental discovery, and hence, the number of potential classes in the sample may have been incorrectly indicated [38, 39]. Thus, three trajectory groups for urban and rural areas were the most acceptable. The rising and remaining-low groups of depression trajectory were observed in urban and rural areas. The main difference was the declining group in urban areas. However, the remaining-high group was observed in rural areas based on the slope of depression score change. Therefore, the three groups in urban areas were rising, remaining-low and declining groups, whereas those in rural areas were rising, remaining-low and remaining-high groups (Fig. 2).

**Table 2:** Model fit indices for longitudinal latent class analysis on CES-D10 over time

Class	AIC	BIC	aBIC	Entropy
Panel A (Urban)				
2(*n*1 = 83%, *n*2 = 17%)	72107.348	72176.508	72141.555	0.824
3(*n*1 = 79%, *n*2 = 11%, *n*3=10%)	71849.745	71937.767	71893.281	0.820
4(*n*1 = 69%, *n*2 = 13%, *n*3 = 13%, *n*4 = 5%)	71521.075	71627.959	71573.941	0.812
Panel B (rural)				
2(*n*1 = 79%, *n*2 = 21%)	134036.208	134111.814	134076.859	0.774
3(*n*1 = 77%, *n*2 = 12%, *n*3=11%)	133792.552	133888.778	133844.289	0.774
4(*n*1 = 63%, *n*2 = 16%, *n*3 = 14%, *n*4 = 7%)	133259.976	133376.823	133322.801	0.758

Table 3 shows the descriptive comparison of baseline characteristics within the urban and rural depression trajectory groups. Physical disability, multimorbidity, gender, marital status, education, family income, smoking and drinking were all significantly different among depression trajectories groups within urban and rural areas. Residential arrangement was not significant within urban and rural depression trajectories. Age and employment were only significant among depression trajectories group within urban areas. No disability, no multimorbidity, female, 50–59 years old, partnered, primary school education, work participation, no smoking, no drinking and living with children were the most common characteristics in the baseline sample in urban and rural areas. The proportions of ADL and IADL in the urban declining group were slightly higher than that in the rising group. The proportion of multimorbidity and absence of spouse in the rural remaining-high group was higher than that in the rising group. Such finding can mean that physical function has a relationship with depression development.

**Table 3:** Descriptive comparison of baseline characteristics within the urban and rural depression trajectory groups

	Urban (*n*=3973)				*P*	Rural (*n*=7139)				
	Total urban	Rising group *n* (%)	Remain low group *n* (%)	Descending group *n* (%)		Total rural	Rising group *n* (%)	Remain low group *n* (%)	Remain high group *n* (%)	*p*
Multimorbidity					<0.01					<0.01
No	2310 (58.14)	166 (42.35)	2005 (62.06)	139 (39.71)		4267 (59.77)	370 (48.88)	3625 (64.39)	272 (36.17)	
Yes	1663 (41.86)	226 (27.65)	1226 (37.94)	211 (60.39)		2872 (40.23)	387 (51.12)	2005 (35.61)	480 (63.83)	
Physical disability					<0.01					<0.01
no limits	3183 (80.12)	249 (63.52)	2738 (84.74)	196 (56.00)		5043 (70.64)	487 (64.33)	4219 (74.94)	337 (44.81)	
IADL-only	323 (8.13)	49 (12.50)	226 (6.99)	48 (13.71)		848 (11.88)	114 (15.06)	626 (11.12)	108 (14.23)	
ADL-only	206 (5.18)	32 (8.16)	142 (4.39)	32 (9.14)		509 (7.13)	65 (8.59)	365 (6.48)	79 (10.51)	
Both	261 (6.57)	62 (15.82)	125 (3.87)	74 (21.14)		739 (10.35)	91 (12.02)	420 (7.46)	228 (30.32)	
Gender					<0.01					<0.01
Male	1823 (45.88)	132 (33.67)	1579 (48.87)	112 (32.00)		3409 (47.75)	267 (35.27)	2920 (51.87)	222 (29.52)	
Female	2150 (54.12)	260 (66.33)	1652 (51.13)	238 (68.00)		3730 (52.25)	490 (64.73)	2710 (48.13)	530 (70.48)	
Age (years)					0.096					<0.01
45–49	843 (21.22)	98 (25.00)	684 (21.17)	61 (17.43)		1405 (19.68)	150 (19.82)	1136 (20.18)	119 (15.82)	
50–59	1499 (37.73)	138 (35.20)	1236 (38.25)	125 (35.71)		2721 (38.11)	271 (35.80)	2179 (38.70)	271 (36.04)	
60–69	1115 (28.06)	111 (28.32)	888 (27.48)	116 (33.14)		2173 (30.44)	248 (32.76)	1645 (29.22)	280 (37.23)	
> 70	516 (12.99)	45 (11.48)	423 (13.09)	48 (13.71)		840 (11.77)	88 (11.62)	670 (11.90)	82 (10.90)	
Marital status					<0.01					<0.01
Partnered	3544 (89.20)	340 (86.73)	2912 (90.13)	292 (83.43)		6329 (88.65)	648 (85.60)	5068 (90.02)	613 (81.52)	
Single	429 (10.80)	52 (13.27)	319 (9.87)	58 (16.57)		810 (11.35)	109 (14.40)	562 (9.98)	139 (18.48)	
Education					<0.01					<0.01
Illiterate	690 (17.37)	101 (25.77)	485 (15.01)	104 (29.71)		2315 (32.43)	311 (41.08)	1661 (29.50)	343 (45.61)	
≤ Primary school	1514 (38.11)	171 (43.62)	1193 (36.92)	150 (42.86)		3056 (42.81)	314 (41.48)	2436 (43.27)	306 (40.69)	
Middle school	1188 (29.90)	90 (22.96)	1029 (31.85)	69 (19.71)		1376 (19.27)	107 (14.13)	1182 (20.99)	87 (11.57)	
≥ High school	581 (14.62)	30 (7.65)	524 (16.22)	27 (7.71)		392 (5.49)	25 (3.30)	351 (6.23)	16 (2.13)	
Employment					0.429					<0.01
Yes	2250 (56.63)	210 (53.57)	1839 (56.92)	201 (57.43)		5834 (81.72)	610 (80.58)	4661 (82.79)	563 (74.87)	
No	1723 (43.37)	182 (46.43)	1392 (43.08)	149 (42.57)		1305 (18.28)	147 (19.42)	969 (17.21)	189 (25.13)	
Family income					<0.01					<0.01
1 (lowest)	797 (20.06)	110 (28.06)	595 (18.42)	92 (26.29)		1448 (20.28)	169 (22.32)	1113 (19.77)	166 (22.07)	
2	800 (20.14)	110 (28.06)	589 (18.23)	101 (28.86)		1410 (19.75)	149 (19.68)	1051 (18.67)	210 (27.93)	
3	788 (19.83)	74 (18.88)	643 (19.90)	71 (20.29)		1426 (19.97)	155 (20.48)	1091 (19.38)	180 (23.94)	
4	794 (19.98)	63 (16.07)	677 (20.95)	54 (15.43)		1428 (20.00)	152 (20.08)	1138 (20.21)	138 (18.35)	
5 (highest)	794 (19.98)	35 (8.93)	727 (22.5)	32 (9.14)		1427 (19.99)	132 (17.44)	1237 (21.97)	58 (7.71)	
Smoke					0.015					<0.01
Yes	1109 (27.91)	97 (24.74)	933 (28.88)	79 (22.57)		2304 (32.27)	204 (26.95)	1934 (34.35)	166 (22.07)	
No	2864 (72.09)	295 (75.26)	2298 (71.12)	271 (77.43)		4835 (67.73)	553 (73.05)	3696 (65.65)	586 (77.93)	
Drink					<0.01					<0.01
Yes	1294 (32.57)	91 (23.21)	1120 (34.66)	83 (23.71)		2373 (33.24)	192 (25.36)	1997 (35.47)	184 (24.47)	
No	2679 (67.43)	301 (76.79)	2111 (65.34)	267 (76.29)		4766 (66.76)	565 (74.64)	3633 (64.53)	568 (75.53)	
Living arrangement					0.135					0.167
Yes	3626 (91.27)	355 (90.56)	2961 (91.64)	310 (88.57)		6362 (89.12)	682 (90.09)	5024 (89.24)	656 (87.23)	
No	347 (8.73)	37 (9.44)	270 (8.36)	40 (11.43)		777 (10.88)	75 (9.91)	606 (10.76)	96 (12.77)	

Table 4 shows the results of multinomial logistic regression analysis between independent variables of baseline and depression trajectory groups within urban and rural areas. A common phenomenon, respondents with multimorbidity were more likely to increase in urban and rural areas, but those in rural locations were also more likely to retain high depression scores, given the rising trajectory group as reference. As for physical disability, with reference to the rising group, those who were physically disabled were more likely to show increased depression scores in urban and rural areas, and the scores of those who need help in ADL and IADL were more likely to continue to be higher in rural areas but may decrease in urban areas. These findings mean that physical function, including multimorbidity and physical disability, are risk factors for depression development in rural areas. The more chronic diseases and difficulties in ADL or IADL, the higher their risk to develop into high-score depression.

**Table 4:** Multinomial logistic regression analysis between independent variables of baseline and depression trajectory groups within urban and rural areas, respectively

	Urban		Rural	
	Remaining low vs. rising group (RRR)	Declining vs. rising group (RRR)	Remaining low vs. rising group RRR)	Remaining high vs. rising group(RRR)
Multimorbidity (ref: no)	0.511***	1.068	0.565***	1.419***
	(0.408, 0.641)	(0.787, 1.449)	(0.482, 0.661)	(1.147, 1.757)
Physical disability (ref: No limits)				
Only IADL	0.547***	1.243	0.744**	1.256
	(0.386, 0.774)	(0.794, 1.946)	(0.593, 0.933)	(0.928, 1.700)
Only ADL	0.515***	1.245	0.758*	1.554**
	(0.339, 0.782)	(0.731, 2.119)	(0.569, 1.010)	(1.081, 2.232)
Both	0.269***	1.498*	0.685***	3.087***
	(0.189,0.383)	(0.994,2.259)	(0.529,0.888)	(2.296,4.151)
Gender (ref: male)	0.637***	1.078	0.578***	1.228
	(0.470, 0.863)	(0.716, 1.624)	(0.462, 0.722)	(0.908, 1.660)
Age (years, ref: 45–49)				
50–59	1.496***	1.480*	1.157	1.094
	(1.120, 1.999)	(0.985, 2.223)	(0.931, 1.440)	(0.809, 1.480)
60–69	1.694***	1.748**	1.028	1.061
	(1.238, 2.317)	(1.139, 2.683)	(0.818, 1.291)	(0.777, 1.447)
≥ 70	2.154***	1.768**	1.254	0.769
	(1.424, 3.257)	(1.017, 3.076)	(0.928, 1.694)	(0.513, 1.185)
Marital status (ref: yes)	1.018	1.274	0.739**	1.135
	(0.726, 1.429)	(0.832, 1.950)	(0.584, 0.935)	(0.847, 1.521)
Education (ref: illiterate)				
Primary school	1.133	0.936	1.224**	1.026
	(0.845, 1.518)	(0.644, 1.361)	(1.021, 1.467)	(0.809, 1.301)
Junior school	1.571***	0.879	1.463***	1.093
	(1.114, 2.215)	(0.554, 1.393)	(1.134, 1.887)	(0.768, 1.555)
Senior school and above	2.023**	1.031	1.730**	0.930
	(1.269, 3.225)	(0.548, 1.943)	(1.112, 2.690)	(0.477, 1.813)
Employment (ref: yes)	1.021	0.720**	1.181	1.056
	(0.806, 1.293)	(0.524, 0.988)	(0.959, 1.456)	(0.810, 1.378)
Family income (ref: lowest)				
2	0.999	1.096	1.066	1.454**
	(0.740, 1.348)	(0.740, 1.622)	(0.839, 1.356)	(1.070, 1.974)
3	1.383*	1.293	1.024	1.225
	(0.996, 1.921)	(0.835, 2.002)	(0.807, 1.301)	(0.898, 1.672)
4	1.451**	1.223	1.005	1.061
	(1.024, 2.056)	(0.758, 1.972)	(0.789, 1.280)	(0.767, 1.468)
5 (highest)	2.633***	1.339	1.186	0.545***
	(1.734, 3.998)	(0.751, 2.388)	(0.921, 1.528)	(0.369, 0.804)
Smoke (ref: yes)	1.334*	1.127	1.176	1.146
	(0.982, 1.811)	(0.745, 1.704)	(0.944, 1.465)	(0.848, 1.548)
Drink (ref: yes)	0.764*	0.889	0.838*	0.856
	(0.571, 1.022)	(0.600, 1.317)	(0.688, 1.021)	(0.656, 1.118)
Living arrangement (ref: yes)	0.825	1.258	1.074	1.231
	(0.567, 1.200)	(0.780, 2.028)	(0.830, 1.390)	(0.887, 1.710)
Cons	25.245***	0.372	34.8085***	0.177***
	(9.977, 63.877)	(0.107, 1.296)	(17.422, 69.546)	(0.069, 0.449)
Obs	3973 0.097		7139 0.085	
Pseudo R^2^				

95% confidence interval are indicated within parenthesis

***p < 0.01, **p < 0.05, *p < 0.1

The association between gender and depression trajectories was consistent between urban and rural areas, that is, female’s depression scores are more likely to rise than that of males. The association between age, marital status, education and depression trajectories differed between urban and rural areas. The CES-D10 scores of the respondents aged 50–59, 60–69 and >70 years from urban areas were more likely to remain low or decline compared with those aged 45–49 years, whereas this association was not significant for respondents from rural areas. The CES-D10 scores of respondents without spouse in rural areas were more likely to rise, whereas the scores were not significantly different between the single and partnered respondents in urban areas. Compared with the uneducated respondents, the CES-D10 scores of the rural respondents who were educated in primary school, junior school, senior school and above were more likely to be lower. Moreover, only the respondents in urban areas who were educated in junior school or senior school and above had significantly lower CES-D10 scores. These results imply that depression development is affected by different factors for urban and rural areas and is thus worthy of further discussion.

## Discussions

In this study, although depression development was divided into three groups in urban and rural areas, trajectories between these locations are different. A significantly declining group was observed in urban areas but not in rural areas. Respondents with multimorbidity were more likely to increase for both urban and rural areas, and the respondents in rural locations were also more likely to retain high depression scores in comparison with the rising group. Similarly, those who were physically disabled in urban and rural area were more likely to achieve high CES-D10 scores. Respondents needing help in ADL and IADL in urban areas were more likely to have declining depression score, whereas the respondents in rural areas were more likely to retain high depression score. Additionally, the association between age, marital status, education and depression trajectories differed between urban and rural areas. Therefore, depression develops differently in urban and rural areas, and the related factors also vary.

The quality of life, uneven allocation of resources in urban and rural areas and social participation of respondents may be potential factors for the unparalleled depression trajectories in urban and rural areas.

The health level influenced by physical health status can be the main and direct reason for depression development. The adverse effects of multimorbidity and physical disability on the health level may increase the CES-D10 scores of the responders [40]. Chronic disease is a condition in which a disease lasts for a prolonged period and is difficult to cure; the causes of chronic diseases are difficult to determine. This condition means that patients will have multimorbidity during the three waves and can experience a continuing adverse effect on their physical health, which may cause respondents with multimorbidity to maintain low CES-D10 scores. Several studies have shown evidence that physical disability will adversely affect the patient’s health level, which further affects the patient’s mental health [41]. Moreover, respondents with physical disability face difficulty to completely recover, similar to those with a chronic disease. Hence, respondents with disabilities with extremely low CES-D10 scores at baseline may rise eventually. No difference was recorded between urban and rural areas with regard to the increase in depressive symptoms, which indicates that the association of multimorbidity and depression trajectories is universal and non-specific.

The long-term uneven distribution of social resources in urban and rural areas may explain that the depression scores of rural patients with multimorbidity remain high, and the older ones in urban areas maintain a low or downward depression scores. With regard to health resources, although China’s rural medical system has been greatly improved, a large gap still exists in the allocation of medical resources between urban and rural residents. The number of health technicians per 1000 individuals in urban and rural China was 7.15 and 4.15 in 2009, respectively, and the values totalled 10.91 and 4.63 in 2018. The number of hospital beds per 1000 individuals in urban and rural areas was 5.94 and 2.60 in 2010 and 8.70 and 4.56 in 2018, respectively [42, 43]. In the long run, the distribution of healthcare resources between urban and rural areas will become more unbalanced. On the other hand, the average reimbursement rate of health insurance for rural residents is lower than that for urban residents, and rural residents encounter higher out-of-pocket costs, which limits the access of rural residents to mental healthcare services [44]. The long-term uneven distribution of healthcare resources and the differences in compensation ratio between urban and rural medical insurance are likely responsible for the additional obstacles encountered by rural patients in accessing quality medical services. This condition may cause the CES-D10 scores of rural patients with multimorbidity to increase for a prolonged period. As for other social benefits, many cities have implemented social benefits for the elderly population, such as free bus rides and regular free medical examinations [22]. This situation may ease the life of urban elderly and may cause the CES-D10 scores of the elderly population in urban areas to remain low and decline.

The difference in social participation between urban and rural areas may lead to an abnormal result of this study, that is, the CES-D10 scores of respondents needing help in ADL and IADL in urban areas were more likely to decline, whereas those of respondents in rural areas were more likely to remain high. Compared with rural areas, urban areas are more likely to provide standardised and high-quality care services for disabled individuals, which alleviates the physical and psychological pain caused by severe disability to a certain extent [45]. Moreover, urban recreational activities are more abundant, whereas rural recreational activities are relatively fewer and are of a gambling nature [46, 47]. This condition may provide disabled patients in urban areas with more opportunities for positive and beneficial social participation and thereby lift their spirit.

Urban and rural women generally participate in the social division of labour, which may be the reason why there is no difference in the depression locus between urban and rural women. The Chinese government has implemented the sunshine project and held many "women's schools" to improve the quality of the female labour force [48]. All these measures have promoted women's employment, especially in rural areas. Extensive employment may make urban and rural women face less different social environment, which may have a similar impact on women's mental health.

The environmental conditions of social participation–social networks are also different, and such situation may be an underlying factor that prompts depression scores to rise in rural respondents without spouses. The social networks of urban residents have contact with people from different backgrounds with weak ties, but the degree of intimacy is low. However, the traditional kinship and neighbourhood relationship are still the main social relations in rural China. The social network among rural residents is limited but strong and stable [49, 50]. As a result, information that a certain individual lacks a spouse may spread fast and become a part of public awareness for a long time within the rural social networks and be perceived negatively. Hence, the social network in a countryside maybe likely less friendly toward individuals without a spouse.

The specific job types of social participation may lead to urban–rural differences in the relationship between education level and depression trajectory. Unlike individuals in rural areas, who are mainly engaged in agriculture and manual labour, a higher proportion of individuals in urban areas are engaged in intellectual activities [51] indicating the demand for a higher education level. An individual with primary school education level only may face difficulties in finding a secure and comfortable life in the city. Hence, individuals with primary school education can maintain a low CES-D10 scores only in rural areas.

## Limitations

Firstly, prior to obtaining baseline measurements, reliable data regarding a respondent’s early history of depression remained unavailable. Therefore, given the limited data, our study only included the result of three waves. Longer longitudinal studies can be conducted when data become available in the future.

Secondly, chronic diseases are self-reported, which results in the underestimation of their association with depression trajectory. The data on chronic diseases were obtained from respondents through a self-reported questionnaire, but no objective medical records were used. Self-reported data may cause underestimation of research results.

Moreover, this study mainly included a family survey and excluded high-risk groups, such as those who were hospitalised with major physical diseases and were unable to recover. However, this condition also suggests that the results obtained were relatively robust.

## Conclusions

The trajectories of depression are different in urban and rural China. Multimorbidity, physical disability, age, marital status and education level have different relationships with the development of depression in urban and rural areas and quality of life. The uneven allocation of resources in urban and rural areas and social participation of respondents may be potential factors. The quality of life should be improved for those with multimorbidity and disabilities by enhancing the level and quality of medical and nursing services to control depression dynamically for a long-term. The allocation of resources needs changes to keep the balance between rural and urban areas. Integrating urban and rural welfare systems (medical insurance, disability care and medical assistance) can be one of the targets. Social participation, especially for the rural elderly, must be promoted by holding recreational activities in rural areas.

## Data Availability

Our dataset is available on http://charls.pku.edu.cn/zh-CN/page/data/harmonized_charls.

## Code availability

The code used and/or analysed during the current study are available from the corresponding author on reasonable request.
